# Phytochemicals as Epigenetic Modulators in Chronic Diseases: Molecular Mechanisms

**DOI:** 10.3390/molecules30214317

**Published:** 2025-11-06

**Authors:** Daniel Cord, Mirela Claudia Rîmbu, Marius P. Iordache, Radu Albulescu, Sevinci Pop, Cristiana Tanase, Maria-Linda Popa

**Affiliations:** 1 Medical Doctoral School, Titu Maiorescu University, 040317 Bucharest, Romania; daniel.cord@prof.utm.ro (D.C.); mirela.rimbu@prof.utm.ro (M.C.R.); mariusiordache.neuro@gmail.com (M.P.I.); 2 Faculty of Medicine, Titu Maiorescu University, 031593 Bucharest, Romania; radu_a1@yahoo.com; 3 Ilfov County Clinical Emergency Hospital, 022104 Bucharest, Romania; 4 National Institute for Chemical-Pharmaceutical R&D, 031299 Bucharest, Romania; 5 “Victor Babeș” National Institute of Pathology, 050096 Bucharest, Romania; spop@ivb.ro; 6 Department of Cell Biology and Histology, “Carol Davila” University of Medicine and Pharmacy, 050474 Bucharest, Romania; maria_lindabv@yahoo.com

**Keywords:** phytochemicals, antioxidant activity, epigenetic mechanisms, anti-inflammatory activity, chemoprevention

## Abstract

Phytochemicals are plant-derived bioactive compounds with antioxidant, anti-inflammatory, and epigenetic modulatory effects that may contribute to the prevention and management of chronic diseases. This review synthesizes recent evidence on the molecular mechanisms through which phytochemicals influence oxidative stress, inflammatory signaling, and epigenetic regulation. A targeted literature search of the PubMed and Web of Science databases (2015–2025) identified over 400 experimental and review studies investigating phytochemicals with documented antioxidant and epigenetic activities. Eligible articles were selected based on relevance to oxidative stress, inflammation, and DNA or histone modification pathways in chronic diseases. Data were qualitatively analyzed to highlight mechanistic links between redox balance, transcriptional regulation, and disease modulation. The results indicate that several phytochemicals, including hesperidin, phloretin, lycopene, and silybin, modulate signaling cascades—NF-κB, Nrf2, and PI3K/Akt—while also influencing DNA methylation and histone acetylation to restore gene expression homeostasis. Despite strong in vitro and in vivo evidence, translation to clinical practice remains limited by low bioavailability, lack of standardized formulations, and insufficient human trials. Future research should prioritize integrative study designs linking molecular mechanisms to clinical endpoints. Understanding the epigenetic actions of phytochemicals may guide the development of nutraceutical strategies for chronic disease prevention.

## 1. Introduction

Phytochemicals are plant secondary metabolites produced as defense molecules against pathogens and environmental stress factors [1]. They display extensive chemical diversity, ranging from low-molecular-weight compounds, such as phenolic acids, to complex structures, such as proanthocyanidins and ellagitannins [2,3]. These naturally occurring compounds possess antiviral, antifungal, and antibiotic properties, as well as photoprotective functions, contributing to plant metabolism and survival under biotic and abiotic stress [4].

For centuries, plant-derived treatments have been used to alleviate acute and chronic diseases, including diabetes, depression, hypertension, hypercholesterolemia, insomnia, and inflammation [5,6]. According to the World Health Organization, nearly 70–80% of the global population still relies on traditional medicine, mainly based on plant extracts [7].

Recent scientific evidence indicates that bioactive compounds from fruits, vegetables, and medicinal plants are biocompatible with cellular and molecular targets and generally less toxic than synthetic drugs [8,9]. Preclinical studies have shown that many phytochemicals with anti-inflammatory, antioxidant, and antiproliferative properties can prevent cancer initiation and progression by activating antioxidant enzymes and triggering apoptosis [10,11]. Moreover, phytochemicals act as substrates, cofactors, or inhibitors in biochemical reactions, as ligands that activate or block receptors, scavengers of reactive oxygen species, and enhancers of nutrient absorption and stability [10,12,13].

There is increasing evidence that chronic diseases such as cancer, metabolic, neurodegenerative, and autoimmune disorders are triggered by epigenetic dysregulation [14]. Epigenetic alterations may result from exposure to environmental factors, including pollutants, radiation, and lifestyle habits such as alcohol consumption, poor nutrition, smoking, and lack of physical activity [15]. Epigenetics refers to heritable changes in gene expression that occur without alteration of the DNA sequence, mainly mediated by DNA methylation, histone modifications, and non-coding RNAs [16,17].

Bioactive dietary compounds can induce protective epigenetic modifications that influence gene expression and health outcomes throughout life. Early identification and correction of epigenetic abnormalities using natural bioactive compounds represent a promising preventive approach [18].

Therefore, understanding the molecular mechanisms through which plant-derived compounds exert antioxidant and anti-inflammatory effects is essential to clarify their impact on human health [19]. This review focuses on the role of phytochemicals as natural bioactive molecules that modulate molecular and epigenetic mechanisms involved in the prevention and management of chronic diseases. A targeted literature search of the PubMed and Web of Science databases (2015–2025) identified over 400 experimental and review studies investigating phytochemicals with documented antioxidant/anti-inflammatory and epigenetic activities. Eligible articles were selected based on relevance to oxidative stress, inflammation, and DNA or histone modification pathways in chronic diseases. Data were qualitatively analyzed to highlight mechanistic links between redox balance, transcriptional regulation, and disease modulation.

## 2. Epigenetic Dysregulation in Chronic Diseases

Over the past decades, numerous studies have underscored the pivotal role of altered expression and activity of epigenetic regulatory proteins in the initiation and progression of various chronic diseases [20].

Epigenetic mechanisms regulate gene expression by modifying DNA, histones, and RNA. For example, the transformation of a condensed chromatin region (heterochromatin), which is transcriptionally inactive, into euchromatin, a transcriptionally active structure, is achieved by adding methyl or acetyl groups to histone proteins. The addition of these chemical radicals, along with the gene’s promoter demethylation, can loosen the chromatin structure, making DNA more accessible to the transcriptional machinery [21].

Enzymes that regulate epigenetic modifications are categorized into “writers,” “erasers,” “readers,” and “remodelers” based on their functions [22]. Writers are adding the specific chemical groups on DNA or RNA bases or histone’s amino acids, whereas erasers are acting in reverse by removing these modifications. For instance, DNA methyltransferase (DNMT) catalyzes the addition of methyl groups to form 5-methylcytosine (m5C) in a gene’s promoter DNA sequence [23]. In contrast, the ten-eleven translocation (TET) enzymes initiate the DNA demethylation process by converting m5C into other derivatives, such as 5-hydroxymethylcytosine (5hmC) [24]. Typically, transcriptionally active genes exhibit lower methylation levels, whereas inactive genes or those with lower expression levels are heavily methylated. Readers are proteins that recognize and bind to these chemical modifications, such as the methyl-CpG-binding domain (MBD), which recognizes 5mC [25]. The reader proteins dynamically influence chromatin status and, by recruiting or collaborating with other protein complexes, regulate gene expression [26].

Remodelers such as the SWI/SNF family, which possess DNA-stimulated ATPase activity, play a crucial role in nucleosome disruption and chromatin accessibility. They achieve this by disrupting nucleosomes and either moving or removing them from key regulatory regions, such as enhancers and promoters, thereby influencing gene expression [26]. Furthermore, there are unique classes of epigenetic regulators, represented by non-coding RNAs (ncRNAs), which directly bind to various genomic regions or specific RNA sequences to modulate gene expression [27].

The long non-coding RNAs (lncRNAs) have a variety of functions and, importantly, can interact with proteins, DNA sequences, and RNAs, regulating the epigenetic machinery directly or indirectly by affecting gene expression and chromatin organization [28,29].

When epigenetic regulators, either epigenetic enzymes or ncRNAs expression, become imbalanced—by being excessively active or insufficiently functional—they disrupt cellular homeostasis and could induce the development of diseases [30]. Consequently, investigating these individual epigenetic alterations, such as DNA methylation, histone modifications, and dysfunctional expression of ncRNAs, represents a promising opportunity for chronic disease therapy, fueling the development of targeted treatments [31,32]. For example, abnormal epigenetic mechanisms are present at various stages of cancer development, including initiation, progression, invasion, migration, and chemotherapy resistance. DNA methylation patterns are changed with hypermethylation of specific tumor suppressors and hypomethylation of oncogene promoters, which drives tumor development [33]. The silencing of tumor suppressor and DNA repair genes disrupts normal cell proliferation and differentiation, fostering the malignant phenotype of tumor cells [34]. Moreover, DNA methylation loss in oncogene promoter regions and extensive demethylation of DNA repetitive sequences compromise chromatin stability, facilitating tumor development [35].

Metabolic disorders, physiological conditions, pre-existing genetic mutations, and exposure to environmental factors, such as dietary patterns, can significantly influence the epigenome [36]. Several studies have shown that in mice fed a high-fat diet, hypermethylation of the promoters of genes such as Rac family small GTPase (Rac1), PPARα, and others is linked to retinal damage and microangiopathy in diabetic retinopathy models [21,37].

Diet-induced epigenetic modifications can affect future generations, heightening their susceptibility to metabolic diseases, including diabetes, non-alcoholic fatty liver disease, and others. Furthermore, diet-related epigenetic changes have been observed to impact the function of epigenetic regulators, enzymes, and their cofactors, such as TET enzymes and α-ketoglutarate (α-KG) derived from the tricarboxylic acid (TCA) cycle, in metabolic disorders [20,38]. This disruption further impairs epigenetic regulation and accelerates disease progression.

Thus, the reversible nature of epigenetic modifications that regulate enzyme activation or inhibition may serve as promising targets and valuable tools for understanding cellular and biological processes.

### 2.1. Oxidative Stress Impacting Epigenetic Mechanisms

The most studied property of plant-derived natural compounds is their antioxidant capacity. Scavenging reactive oxygen species (ROS) is thought to be an effective way to reduce oxidative stress in cells.

ROS are represented by free radicals such as hydrogen peroxide, hydroxyl radical (OH^−^), peroxy radical (ROO^−^), superoxide anion radical (O_2_^−^), and singlet oxygen, among others, which are necessarily produced in the human body as a result of normal physiological processes occurring, and also by exposure to environmental stressors. Oxidative stress arises when the balance between ROS levels is disrupted and the cellular antioxidant defense system is overwhelmed [39].

Oxidative stress is a key modulator of the cellular environment, often altering gene expression through epigenetic modifications. The imbalance between pro-oxidant species and antioxidant defenses can lead to changes in DNA methylation patterns, histone modifications, and non-coding RNA expressions, which collectively impact chromatin structure and gene transcription. For instance, oxidative stress induces the formation of 8-oxo-2′-deoxyguanosine (8-oxo-dG), a DNA lesion that recruits repair machinery but also affects the local methylation status, potentially silencing tumor suppressor genes or activating oncogenes. Such oxidative DNA damage has been linked to disrupted DNMT activity and subsequent global DNA hypomethylation [40].

Multiple lines of scientific evidence have linked the oxidative stress process to pathophysiology associated with various diseases, including cancer [41,42]. ROS can effectively induce DNA damage through nucleotide alterations, DNA strand breakage, and mutations in the genome of normal cells, thereby promoting further disease-related transformation. Moreover, ROS can activate transcription factors, including NF-κB, cyclooxygenases (COX), p53, HIF-1α, PPAR-γ, β-catenin/Wnt, and Nrf2, leading to the expression of over 500 genes, including growth factors, pro- and anti-inflammatory cytokines, and cell cycle regulatory molecules. [43,44]. Moreover, Nrf2, a crucial transcription factor that acts as a master regulator of cellular defense mechanisms against oxidative stress, electrophiles, and other harmful substances, can influence epigenetic mechanisms by modulating histone acetylation and deacetylation [45]. This activity reshapes the cell’s epigenetic landscape, impacting gene expression in response to oxidative stress [46].

In addition, the ROS can influence the DNA methylation pattern by directly oxidizing the catalytic site of DNMTs or by reducing the availability of the DNMT cofactor S-adenosyl methionine, SAM [46]. Cellular ROS levels themselves have been suggested to be the subject of epigenetic modulation. Thereby, ROS-generating systems in mitochondria, including various subunits of the NOX complex, as well as antioxidant enzymes such as SODs and catalase, have been reported to be epigenetically regulated by distinct mechanisms [47]. ROS can modulate the activity of other epigenetic regulators such as histone acetyltransferases (HATs) and histone deacetylases (HDACs), influencing the histone acetylation epigenetic mechanism and thus, such as histone acetyltransferases (HATs) and histone deacetylases (HDACs), thereby altering the histone acetylation epigenetic mechanism and inducing dysregulated gene expression in cancer and cardiovascular disorders [46].

Phytochemicals are highly bioactive molecules; however, a particular class, namely phenolics (phenolic acids and flavonoids), is the most abundant and exhibits the highest antioxidant activity in the cellular environment [47,48].

**Figure 1:**
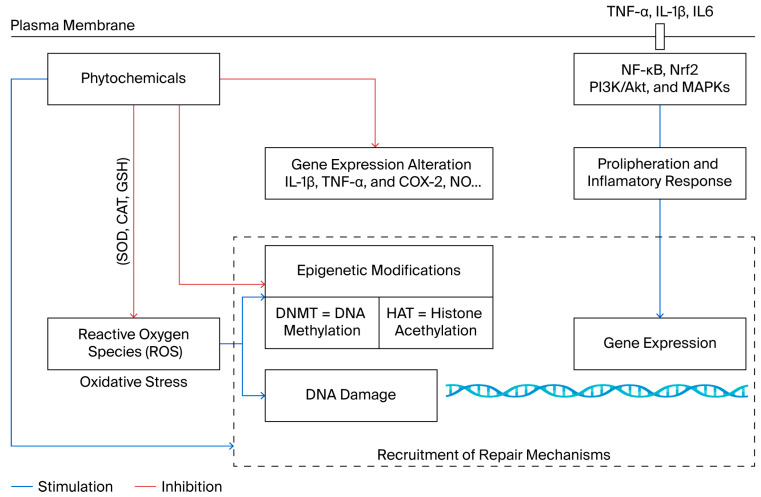
Phytochemical-mediated regulation of redox balance, inflammation, and epigenetic mechanisms: Phytochemicals play multifaceted roles in preserving cellular homeostasis by coordinating redox balance, inflammatory signaling, and epigenetic control. By scavenging reactive oxygen species (ROS) and activating antioxidant defense systems such as the Nrf2/ARE pathway, phytochemicals enhance the expression of enzymes including superoxide dismutase (SOD), catalase (CAT), and glutathione peroxidase (GPx). Simultaneously, they inhibit pro-inflammatory mediators—NF-κB, COX-2, iNOS, IL-1β, and TNF-α—attenuating cytokine overproduction and tissue damage. At the epigenetic level, phytochemicals modulate DNA methyltransferases (DNMTs), histone deacetylases (HDACs), and histone acetyltransferases (HATs), restoring physiological gene expression patterns by reversing hypermethylation of tumor suppressor genes and promoting histone acetylation. Through these convergent molecular actions, phytochemicals link oxidative and inflammatory responses to chromatin remodeling and transcriptional reprogramming, supporting chemopreventive and therapeutic effects in chronic diseases.

Different mechanisms underlie the antioxidant capacity of phytochemicals, including free-radical scavenging, hydrogen atom donation, chelation of ferric or cupric ions, single-oxygen quenching, and acting as a substrate for superoxide and hydroxyl radicals (Figure 1) [10,49]. The antioxidant activity of phytochemicals also relies on their capacity to inhibit essential enzymes responsible for the endogenous production of reactive oxygen species (ROS), such as COXs, cytochrome P450 (CP450), lipoxygenase (LO), NADPH oxidase (NOX), nitric oxide synthases (NOSs), and xanthine oxidase (XO) [50]. The production of these enzymes is increased during inflammatory conditions; thus, inhibiting them can prevent ROS overproduction, thus mitigating the associated cellular damage and inflammation [51]. Since mediators of inflammation play a key role in various pathologies, such as cancer, metabolic, and neurodegenerative disorders, natural bioactive compounds might exert both antioxidant and anti-inflammatory activities [51].

According to recent scientific reports, the main molecular mechanism by which phytochemicals reduce cellular oxidative stress is by activating the Nrf2 pathway, which boosts antioxidant defense systems and diminishes inflammation [52,53]. Phytochemicals activate the Nrf2 pathway to combat oxidative stress primarily by modifying Keap1, the central negative regulator of Nrf2, or by promoting phosphorylation of Nrf2. Under normal conditions, Nrf2 is bound to Keap1 in the cytoplasm, which leads to its ubiquitination and degradation [54]. When phytochemicals induce oxidative or electrophilic stress, they cause conformational changes in Keap1 or directly modify it, leading to the release of Nrf2. Freed Nrf2 then translocates to the nucleus, where it forms heterodimers with Maf proteins and binds to antioxidant response elements (AREs) in the DNA. This binding initiates transcription of various antioxidant and detoxification genes, such as heme oxygenase-1 (HO-1), glutathione S-transferase (GST), and NAD(P)H: quinone oxidoreductase 1 (NQO1), which enhance cellular antioxidant defenses and reduce oxidative damage [55].

At the same time, phytochemicals influence epigenetic processes, acting as epigenetic modulators that primarily inhibit key epigenetic enzymes, including DNMTs, HDACs, HATs, and BETs [56].

Moreover, phytochemicals can donate the methyl group directly as a co-substrate in DNA, RNA, or histone methylation or indirectly by affecting the methyl pool [23].

As antioxidants, anti-inflammatory molecules, and epigenetic modulators, the phytochemicals represent valuable candidates for disease prevention and treatment through coordinated regulation of redox balance, the inflammatory process, and epigenetic mechanisms (Figure 1).

### 2.2. Epigenetic Dysregulation in the Inflammation Process

Inflammation is a highly dynamic and tightly regulated biological response, essential for defending the body against infections, injuries, and other harmful stimuli [57]. However, when the mechanism of controlling inflammation becomes dysregulated, the results can be chronic inflammatory states that contribute to a wide range of diseases, from autoimmune disorders to cancer and cardiovascular pathologies [58]. Epigenetic mechanisms are increasingly recognized for their essential role in inflammation. They act as modulators of normal inflammatory responses by fine-tuning the protein expression and also as drivers of pathological inflammation when disrupted [59].

Epigenetic dysregulation is increasingly recognized as a critical factor in modulating inflammation and immune responses. DNA methylation plays a dual role, either suppressing or activating key pro-inflammatory genes. Aberrant activity of DNA methyltransferases (DNMTs) can lead to inappropriate methylation patterns that sustain chronic inflammation by silencing anti-inflammatory genes. For example, DNMT3B has been shown to hypermethylate promoter regions of tumor suppressor genes, thereby contributing to sustained inflammatory signaling [40]. Recent studies indicate that DNMT1-mediated methylation of IL-10 and SOCS3 promoters also maintains pro-inflammatory macrophage polarization, thus reinforcing chronic inflammatory loops [60].

During an inflammatory response, immune cells such as macrophages, dendritic cells, and lymphocytes undergo rapid changes in gene expression. This transcriptional reprogramming is orchestrated in part by epigenetic modifications, which enable these cells to rapidly activate or repress large sets of genes in response to signals such as cytokines, pathogen-associated molecular patterns (PAMPs), or damage-associated molecular patterns (DAMPs) [61,62,63]. Such transcriptional shifts are supported by histone acetylation at promoters of TNF-α, IL-1β, and IL-6, mediated by p300/CBP histone acetyltransferases, which cooperate with NF-κB to enhance transcriptional output during acute inflammation. Conversely, HDAC3 recruitment by NF-κB p50 homodimers helps reduce inflammatory signaling [64]. Epigenetic mechanisms not only determine which genes are expressed, but also regulate the intensity of cellular response to inflammatory stressors [65].

Multiple epigenetic modifications may occur in a wide range of diseases associated with or related to inflammatory processes. For instance, the most investigated class of diseases, cancers, are often associated with epigenetic deregulation, which can occur in tumoral stroma or in immune cells. Epigenetics encompasses a series of reversible modifications that do not involve gene mutations and are strongly modulated by the internal environment, particularly that located close to tumoral processes. In this way, the pro-inflammatory environment surrounding the tumor can induce epigenetic modifications [66,67]. For example, persistent IL-6/STAT3 signaling within the tumor microenvironment induces DNMT1 and EZH2 expression, thereby reinforcing the epigenetic repression of tumor suppressor genes and maintaining a pro-oncogenic inflammatory state [68].

Yang et al. [69] analyzed the implications of epigenetic modifications—DNA methylation, chromatin remodeling, regulation of non-coding RNAs, and histone changes—during the progression from chronic inflammation to colorectal cancer. The study also outlined the accelerating effects of such changes on the activation of cancer signaling pathways. Among these, the NF-kB and STAT3 pathways appear to be of notable impact. A similar analysis was provided by Pandareesh et al. for prostate cancer, with an almost identical distribution and interaction among epigenetic factors, the tumor environment, and the pro-inflammatory environment [70]. Mechanistically, NF-κB activation enhances histone acetylation at promoters of inflammatory cytokines (IL-8, COX-2), while STAT3 can recruit DNMT1 to hypermethylate SOCS gene promoters, reinforcing a self-sustaining inflammatory state in cancer [71].

When epigenetic control mechanisms malfunction, the balance between pro-inflammatory and anti-inflammatory pathways can be disrupted [61,72]. For instance, aberrant DNA methylation patterns may silence anti-inflammatory genes (e.g., IL-10) or abnormally activate pro-inflammatory ones, such as IL-6 or TNF-α [73,74,75]. Moreover, histone modifications can alter chromatin accessibility and maintain certain genes in a persistently active state, even in the absence of inflammatory stimuli. For example, increased histone acetylation of NF-κB target gene promoters has been linked to sustained inflammation in rheumatoid arthritis and inflammatory bowel disease [76,77,78]. In these contexts, HDAC inhibitors have been shown to restore the balance of histone acetylation and suppress NF-κB-dependent transcription, partly by enhancing the acetylation and stability of IκBα, thereby preventing NF-κB nuclear translocation [79].

Notably, non-coding RNAs, such as miR-155 or miR-21, are often overexpressed in chronic inflammation and can modulate immune cell differentiation, cytokine production, and apoptosis resistance [80,81]. The eraser proteins HDACs are inhibited, leading to increased histone acetylation, opening chromatin and facilitating the expression of anti-proliferative and regulatory genes like p21 [82,83]. HDAC1 and HDAC2 repression has also been linked to enhanced p21 and GADD45β expression, which attenuate NF-κB-mediated inflammatory transcription [84].

The p21 gene plays a multifaceted role in cellular processes. It acts as a cyclin-dependent kinase (CDK) inhibitor, primarily inhibiting CDK2 and CDK1, which are crucial for cell cycle progression [85]. This inhibition leads to cell cycle arrest, particularly at the G1/S and G2/M transitions, preventing uncontrolled cell division and promoting genomic stability after DNA damage. Beyond cell cycle regulation, p21 also influences the cellular environment by secreting bioactive molecules, contributing to immunomodulatory functions and potentially aiding in the resolution of inflammation [86].

Histone modifications further contribute to inflammation-driven gene expression. Pro-inflammatory cytokines like TNF-α and IL-6 are regulated by dynamic histone acetylation and methylation at their promoter regions, with histone acetyltransferases (HATs) enhancing expression and histone deacetylases (HDACs) serving as brakes on transcription. Natural polyphenols such as resveratrol and quercetin have been shown to modulate these enzymes, reducing pro-inflammatory histone marks while enhancing the expression of anti-inflammatory genes [87]. For example, resveratrol promotes deacetylation of p65/RelA via SIRT1 activation, reducing NF-κB transcriptional activity, while quercetin inhibits p300-mediated H3K27 acetylation at IL-1β promoters [88].

Non-coding RNAs, especially microRNAs (miRNAs), play a pivotal role in fine-tuning inflammation. For instance, miR-146a suppresses NF-κB signaling, a master regulator of inflammation. Under oxidative stress conditions, miR-146a expression can be epigenetically downregulated, leading to sustained inflammation [89]. Additionally, decreased miR-146a expression has been associated with reduced negative feedback on TRAF6 and IRAK1, amplifying NF-κB activity in chronic inflammatory states [90]. Interestingly, dietary polyphenols may also exert protective effects on inflammation by modulating Nrf2, which coordinates antioxidant responses and regulates inflammatory pathways. By activating the Nrf2-ARE pathway, compounds like curcumin, sulforaphane, and EGCG (epigallocatechin gallate) can upregulate antioxidant genes while simultaneously downregulating pro-inflammatory mediators [91]. Curcumin, for example, directly promotes Nrf2 nuclear translocation and enhances HO-1 and NQO1 expression, while simultaneously repressing NF-κB p65 phosphorylation, providing dual antioxidant and anti-inflammatory benefits [92,93]. Moreover, recent evidence indicates that dysregulated Nrf2 signaling contributes to cancer cell survival under oxidative stress, positioning Nrf2 both as a protective and potentially oncogenic factor [94]. Furthermore, recent evidence from a clinical study suggests that polyphenol-rich diets can alter epigenetic markers of aging in immune cells, thereby indirectly influencing inflammation status [91]. Such interventions might slow down immunosenescence and mitigate inflammation-associated disorders.

Moreover, such epigenetic changes can affect not only immune cells but stromal and epithelial cells within the inflamed microenvironment, contributing to persistent tissue remodeling and fibrosis [66].

Another field of major interest is coronary artery disease, where, on the one hand, disease progression involves epigenetic changes, such as histone methylation, phosphorylation, and acetylation, playing key roles. In addition to these, the lncRNAs, miRNAs, and circRNAs are also involved in epigenetic processes and help regulate gene expression in response to environmental factors and lifestyle. Such modifications in the deregulation of genes regulating vascular function, inflammation, lipid metabolism, or oxidative stress [95,96].

Collectively, this epigenetic modification can act as a form of inflammatory memory, locking cells into a pro-inflammatory state even after the initial trigger has passed [62]. This phenomenon is especially evident in chronic diseases such as atherosclerosis, rheumatoid arthritis, and asthma, in which immune cells exhibit persistent activation profiles [97].

A defining feature of epigenetic regulation is the plasticity of epigenetic marks, which are not static and can be influenced by environmental exposures [98]. Factors such as smoking, diet, psychological stress, pollution, infections, and gut microbiota-derived metabolites can all modify the epigenetic landscape [99,100].

For instance, short-chain fatty acids, such as butyrate, produced by commensal bacteria, can inhibit HDACs and thereby alter gene expression in immune cells [101]. Similarly, oxidative stress induced by environmental pollutants can trigger histone modifications that amplify transcription of inflammatory genes [102,103]. These influences suggest that chronic exposure to harmful environmental factors may not only trigger inflammation but may epigenetically fix it into the biology of the tissues [104,105].

Even subtle lifestyle factors, such as circadian rhythm disruption or chronic low-grade stress, may modulate the expression of inflammatory genes through epigenetic routes [106]. These influences suggest that chronic exposure to harmful environmental factors may not only trigger inflammation but also epigenetically fix it into the tissue’s biology [107].

Of interest is also the fact that natural compounds, for instance naringenin and naringin (pharmacologically active in anticancer and cardiovascular diseases, with antioxidant and anti-inflammatory activities), are involved in epigenetic mechanisms, thus broadening the mechanisms supporting the restoration of health [108,109].

Overall, the integration of dietary components, oxidative stress, and epigenetic alterations forms a complex network that modulates inflammation. Targeting these pathways through nutraceuticals offers a promising avenue for managing chronic inflammation-related diseases.

The reversibility of epigenetic marks makes them attractive targets for novel therapeutic strategies. Drugs that inhibit DNMT1 or HDACs are already being explored in cancer therapy, but they show promise in treating chronic inflammatory diseases as well. By reprogramming immune cells epigenetically, either to reduce their inflammatory potential or to restore tolerance, such an intervention could offer a more precise and durable solution than broad-spectrum immunosuppressants [110]. For example, HDAC inhibitors may promote the expression of p21 and other regulatory genes, helping to dampen persistent inflammation. Similarly, DNMT inhibitors could reactivate silenced anti-inflammatory genes or detoxifying enzymes such as GSTP1, restoring homeostasis in chronically inflamed tissues [111].

## 3. Phytochemicals as Antioxidant and Anti-Inflammatory Agents with Epigenetic Modulator Capacities

Chronic inflammation and oxidative stress are fundamental contributors to the pathogenesis of numerous non-communicable diseases, including cardiovascular disorders, cancer, neurodegenerative conditions, diabetes, and autoimmune syndromes. Oxidative stress arises from an imbalance between reactive oxygen species and the antioxidant defense system, which may also induce inflammation by elevating the levels of pro-inflammatory mediators and cytokines. These two pathological states are tightly interconnected and often exacerbate one another, creating a self-perpetuating cycle of tissue damage and immune dysregulation [112].

In recent years, phytochemicals found in fruit, vegetables, herbs, and medicinal plants have gained significant attention for their ability to counteract oxidative and inflammatory damage. These compounds include flavonoids, phenolic acids, terpenoids, alkaloids, and organosulfur compounds, many of which possess strong radical-scavenging capabilities and modulate inflammatory signaling pathways such as NF-κB, Nrf2, PI3K/Akt, and MAPKs (Table 1) [113,114].

Unlike conventional pharmaceuticals, phytochemicals often have multitarget potential, modulating cellular processes simultaneously and often with lower toxicity profiles. For instance, flavonoids enhance antioxidant enzyme activities, such as SOD, CAT, and GSH, while simultaneously inhibiting pro-inflammatory mediators, such as TNF-α, IL-6, and COX-2, through the suppression of transcription factors and kinases [115,116,117]. These mechanisms not only alleviate oxidative stress but also reduce systemic and local inflammation, contributing to disease prevention and adjunctive therapy. Notably, flavonoids represent a structurally diverse class of compounds, including flavonols, flavones, flavanones, and anthocyanidins, each with distinct bioactivities and tissue selectivity [118]. This diversity allows them to modulate a broad range of molecular targets implicated in inflammation, oxidative damage, and metabolic imbalance [119]. The phytochemical multitarget capacity supports their potential for disease prevention and as adjunctive therapy.

At the mechanistic level, NF-κB activation typically involves phosphorylation and degradation of its inhibitor IκBα by the IKK complex, allowing the p65/p50 heterodimer to translocate into the nucleus and transcribe pro-inflammatory genes such as *COX-2*, *iNOS*, and *IL-6* [120,121]. Several phytochemicals, including genistein and phloretin, have been shown to inhibit this phosphorylation cascade, thereby attenuating NF-κB nuclear translocation and cytokine production [122].

Conversely, the Nrf2 pathway acts as a central defense mechanism against oxidative stress [53,123]. Under basal conditions, Nrf2 is sequestered in the cytoplasm by Keap1; oxidative or electrophilic stress disrupts this complex, enabling Nrf2 to translocate into the nucleus and induce transcription of antioxidant and cytoprotective genes such as *HO-1*, *NQO1*, and *GCLC* [53,54,124].

Compounds such as curcumin, artemisinin, and lycopene can activate Nrf2 either by modifying Keap1 cysteine residues or by enhancing Nrf2 stability [125,126].

Recent studies have provided further evidence of curcumin’s role as a potent activator of the Nrf2/ARE pathway [92]. These works highlight curcumin’s ability to promote Nrf2 nuclear accumulation and upregulation of its downstream targets, including *HO-1* and *NQO1*, which together mitigate ROS-induced cellular damage [127]. However, the emerging literature also emphasizes the dual role of Nrf2 in cancer [94]: while transient activation supports antioxidant defense and cytoprotection, chronic or constitutive activation in tumor cells may promote survival, chemoresistance, and metabolic adaptation [126]. Therefore, the biological outcome of Nrf2 activation depends on the cellular and pathological context, underscoring the need for balanced modulation of this pathway [128,129].

Beyond these canonical signaling routes, phytochemicals also modulate epigenetic mechanisms that regulate gene expression without altering DNA sequence [130,131,132,133,134]. Several compounds inhibit DNA methyltransferases (DNMTs) and histone deacetylases (HDACs), thereby reactivating silenced tumor suppressor genes such as *p16*, *p21*, *SOCS3*, and *RARB* [133,134,135]. For instance, lycopene and silibinin have been shown to reduce DNMT1 and HDAC2 expression in hepatic and prostate cell lines, restoring normal histone acetylation patterns and promoting apoptosis [134,136]. Similarly, phloretin and caffeic acid increase histone acetylation and DNA hypomethylation, thereby facilitating transcription of antioxidant and anti-inflammatory genes [137,138].

Table 1 presents the phytochemicals with in vitro-demonstrated antioxidant, anti-inflammatory, and potential epigenetic modulator capacities.

**Table 1:** Example of phytochemicals with antioxidant, anti-inflammatory activities, and epigenetic modulator capacity, investigated in vitro.

Class of Compound	Phytochemicals	Study Design: Cell Culture Type; Phytochemicals Concentration	Key Outcomes			References
			Antioxidant	Anti-Inflammatory	Epigenetic Modulator	
Flavonoids	Hesperidin	MCF-7 (50 µM)	Increases activities of SOD, CAT, GSH; reduces lipid peroxidation; activates Nrf2	Reduces the expression of TNF-α and IL-6	Inhibits DNMT1; hypo-methylation of p16 promoter	[54,55,56]
	Phloretin	RAW 264.7 macrophages A549 cells (25 µM);	Inhibits ROS production and increases GSH activity	Suppresses NF-κB activation and reduces iNOS and COX-2 expression	Inhibits HDAC; increases histone acetylation, p21 expression	[139,140,141,142]
	Genistein	MCF-7, (10 µM) PC3 (25 µM–100 µM), HL-60 cells	Increases antioxidant enzymes; reduces lipid peroxidation	Reduces pro-inflammatory cytokines (IL-6, IL-1β) expression via NF-κB	Inhibits DNMT1, demethylates, and reactivates tumor suppressor genes	[143,144,145,146]
Phenolic acids	Caffeic acid	Caco-2, HeLa cells (20 µM)	Neutralizes free radicals and boosts antioxidant enzymes	Inhibits pro-inflammatory cytokines and NF-κB activation	Inhibits DNMT; DNA hypomethylation; gene reactivation	[147,148,149]
	Coumaric acid	LPS-stimulated RAW 264.7 cells; MDA-MB-231 cells (30 µM)	Free radical scavenger and increases SOD activity	Reduces NO production and COX-2 expression	Inhibits HDAC; increases histone acetylation	[150,151,152]
Terpenoids	Lycopene	HepG2, PC3 cells (10 µM)	Reduces ROS and increases antioxidant enzyme activity	Inhibits COX-2 expression and lowers prostaglandin E2 levels	Inhibits DNMT1 and HDAC2 expression; modulates miR-let-7f-1/AKT2 axis; restores tumor suppressor genes (*p21*, *RARB*, *SOCS3*).	[136,153]
	Silibinin	HepG2, DU145 cells (50 µM)	Increases SOD and CAT activity; reduces lipid peroxidation	Inhibits pro-inflammatory cytokines and NF-κB activation	Inhibits DNMT and HDAC; hypomethylation and histone acetylation	[154,155,156]
	Artemisinin	HeLa cells (50 µM)	Reduces oxidative stress by lowering ROS and activating Nrf2	Inhibits IL-1β, TNF-α, and COX-2 expression	—	[157]
	Geraniol	RAW 264.7 macrophages (50 µM)	Reduces ROS production and increases GSH activity	Suppresses NO, IL-6, and TNF-α production by inhibiting NF-κB	—	[158]
Organo-sulfur com-pounds	Allyl mercaptan	HUVEC, HT-29 cells (15 µM)	Enhances antioxidant enzyme activity and reduces ROS	Inhibits adhesion molecules and reduces vascular inflammation	Inhibits HDAC; increases histone acetylation; gene reactivation	[159,160]

In summary, phytochemicals exert their antioxidant and anti-inflammatory actions through interconnected pathways that encompass the inhibition of NF-κB–driven transcription, activation of the Nrf2/ARE antioxidant response, and epigenetic reprogramming of gene expression. The convergence of these molecular mechanisms provides a comprehensive perspective on their therapeutic potential.

### 3.1. Flavonoids

Hesperidin, a bioactive flavonoid predominantly found in citrus such as oranges and lemons, has demonstrated significant antioxidant and anti-inflammatory properties, particularly in the context of breast cancer [161,162]. In vitro studies on MCF-7 breast cancer cells treated with 50 µM hesperidin showed increased antioxidant enzymes (SOD, CAT, and glutathione) and reduced lipid peroxidation [145]. These effects are attributed to the activation of the Nrf2 signaling pathway, which plays a crucial role in cellular defense against oxidative stress. Furthermore, hesperidin significantly decreased the expression of pro-inflammatory cytokines such as TNF-alpha and IL-6 by inhibiting the pathway, thereby modulating inflammation within the tumor microenvironment [163,164]. These findings underscore hesperidin’s potential as a therapeutic agent managing oxidative stress and inflammation in breast cancer models [123]. Beyond its antioxidant and anti-inflammatory activities, hesperidin also exerts epigenetic modulatory effects. Studies have demonstrated that hesperidin can reduce global DNA methylation levels by inhibiting DNA methyltransferases (DNMTs), particularly in cancer models [165]. In vitro and in vivo cancer models have shown that hesperidin downregulates hypermethylation of repetitive DNA elements, such as KINE-1 and ALU sequences, and modulates microRNA expression, including the upregulation of tumor-suppressive miR-34a and the downregulation of oncogenic miR-221 [166]. These epigenetic modifications contribute to the reactivation of tumor suppressor genes and the inhibition of oncogenic pathways, further supporting hesperidin’s role in cancer and chemoprevention. These findings suggest that hesperidin supports human health through several interconnected mechanisms. By enhancing antioxidant defenses, reducing harmful inflammation, and influencing epigenetic processes that control gene activity, hesperidin shows great promise as a natural compound that could help prevent or support the integrative treatment of cancer.

Genistein is a naturally occurring isoflavone; it is predominantly found in leguminous plants, especially soybeans (*Glycine max*), where it occurs mainly as the glycoside genistin [167]. Genistein exhibits a spectrum of biological activities beyond its well-known estrogenic and anticancer effects, including moderate antioxidant and anti-inflammatory actions, as well as epigenetic mechanisms regulator [168,169]. This compound exerts moderate but significant antioxidant effects, primarily by reducing ROS [39] and enhances the activities of antioxidant enzymes such as SOD, catalase CAT and glutathione peroxidase, largely via the Nrf2 signaling pathway [170]. These effects stabilize redox balance and protect cellular components from oxidative damage, especially under inflammation or tumorigenic conditions. In addition to its redox balancing effects, genistein demonstrated anti-inflammatory activity by downregulating the NF-kB signaling pathway and suppressing the expression of pro-inflammatory cytokines [171] such as TNF-alpha, IL-6, and Il-1ß [172].

Genistein also inhibits the activity of enzymes such as COX-2 and iNOS, thereby further reducing the inflammatory response [173]. This is particularly relevant in chronic diseases where low-grade inflammation plays a pathogenic role in atherosclerosis and arthritis. One of genistein’s most compelling biological features is its ability to modulate epigenetic mechanisms by inhibiting DNMTs, especially DNMT1, thereby reactivating silenced tumor suppressor genes such as p16, RASSF1A, and BRCA1 [174,175]. Recent findings also suggest that genistein can modulate sirtuin activity and potentially interact with TET enzymes, thereby impacting DNA demethylation and metabolic-epigenetic crosstalk [176].

Phloretin, a dihydrochalcone flavonoid predominantly found in apples, has demonstrated notable antioxidant and anti-inflammatory properties. In vitro studies using RAQ 264.7 macrophages treated with 25 µM phloretin have shown a significant reduction in reactive oxygen species (ROS) production and an increase in intracellular GSH levels, indicating its potential to mitigate oxidative stress [177].

Furthermore, phloretin has been shown to suppress nuclear factor kappa B (NF-κB) activation, a pivotal transcription factor in the inflammatory response. This suppression leads to the downregulation of pro-inflammatory enzymes such as inducible nitric oxide synthase (iNOS) and cyclooxygenase-2 (COX-2). Additionally, phloretin modulates the mitogen-activated protein kinase (MAPK) pathways, including extracellular signal-regulated kinase (ERK), c-Jun N-terminal kinase (JNK), and p38 MAPK [178]. These findings are supported by Leet et al., who demonstrated that phloretin attenuates lipopolysaccharide-induced acute lung injury in mice by blocking the NF-kB and MAPK pathways [179]. These pathways are integral to the production of pro-inflammatory mediators [177]. The combined modulation of these signaling pathways by phloretin suggests its potential application in managing chronic inflammatory conditions and cardiovascular diseases [180,181].

In addition to its well-established antioxidant and anti-inflammatory actions, emerging evidence suggests that phloretin may act as an epigenetic modulator. According to recent findings, phloretin can inhibit DNA methyltransferases (DNMTs), enzymes that add methyl groups to DNA and are often involved in the silencing of tumor suppressor genes [40]. By modulating DNA methylation, phloretin may help restore normal gene expression profiles disrupted in various chronic diseases, including cancer. Although further research is needed to clarify its impact on histone modification and microRNA regulation, these initial insights suggest that phloretin’s health benefits extend beyond redox and inflammatory pathways to include epigenetic regulation of gene expression. Altogether, these multiple molecular actions, combining antioxidant, anti-inflammatory, and epigenetic modulatory effects, position phloretin as a promising candidate for managing chronic inflammatory diseases, metabolic disorders, and cancer prevention.

Silybin, a flavonolignan extracted from Silybum marianum (milk thistle), is widely recognized for its hepatoprotective, antioxidant, and anti-inflammatory effects. In vitro studies on HepG2 cells have shown that silybin increases the activity of endogenous antioxidant enzymes, including SOD and CAT, while significantly decreasing lipid peroxidation, thereby alleviating oxidative damage [182,183]. The compound also inhibits NF-Kb activation, the key transcription factor that regulates inflammatory cytokines [184]. As a result, treatment with silybin leads to reduced expressions of TNF-α, IL-6, and iNOS, contributing to a pronounced anti-inflammatory effect. Moreover, it modulates JAK/STAT and TGF-βsignaling pathways, both of which play central roles in liver fibrosis and hepatocarcinogenesis [185].

Beyond its cellular effects, silibinin is under clinical investigation for its antifibrotic and chemoprotective potential, particularly in the context of chronic liver diseases such as non-alcoholic fatty liver disease (NAFLD) and hepatocellular carcinoma (HCC) [186]. Its multifunctional properties, spanning antioxidation, inflammation suppression, and interference with the fibrotic pathway, highlight its promise as a therapeutic agent in hepatic disorders [186].

In addition to these biological effects, silybin has also been shown to exert epigenetic regulatory activities. Silybin modulates the activity of DNA methyltransferases (DNMTs) and histone deacetylases (HDACs), leading to changes in DNA methylation and histone acetylation status in liver cells. These epigenetic modifications reactivate tumor suppressor genes and suppress oncogenic pathways, thereby contributing to their chemopreventive potential in hepatocarcinogenesis. Therefore, the therapeutic role of silybin in liver diseases may also involve epigenetic regulation of gene expression, providing a broader mechanistic basis for its hepatoprotective and anticancer effects.

### 3.2. Carotenoids

Lycopene, a lipophilic carotenoid found in tomatoes and watermelon, acts as a potent scavenger of ROS in HepG2 cells [187]. At a concentration of 10 µM, lycopene reduced ROS levels and increased antioxidant enzyme activity [188]. Additionally, it inhibited COX-2 expression and decreased prostaglandin E2 synthesis, indicating an anti-inflammatory effect. Lycopene also modulates transcription factors such as AP-1 and STAT3, suggesting its potential for liver cancer prevention and for reducing hepatic inflammation [189]. In addition to these well-established effects, lycopene also exerts epigenetic modulatory activity. Recent findings indicate that lycopene can influence DNA methylation patterns by inhibiting DNA methyltransferases (DNMTs). This inhibition may contribute to the reactivation of silenced tumor suppressor genes and the correction of aberrant gene expression associated with carcinogenesis. While further research is needed to fully elucidate its effects on histone modification and non-coding RNAs, these early insights suggest that lycopene contributes to the regulation of gene activity through epigenetic pathways, adding a new layer to its chemopreventive potential. Overall, these findings suggest that lycopene supports liver health through several complementary actions. By reducing oxidative stress, calming inflammation, and influencing gene activity via epigenetic mechanisms, lycopene shows promise in cells and may reduce the risk of liver cancer [40].

### 3.3. Phenolic Compounds

Caffeic acid, a hydroxycinnamic acid prevalent in coffee, fruits, and vegetables, has demonstrated significant antioxidant and anti-inflammatory properties, particularly within intestinal epithelial cells [190,191]. In vitro studies utilizing Caco-2 cells treated with 20 µM caffeic acid have shown a notable reduction in ROS production and an enhancement of endogenous antioxidant enzyme activities, including SOD, CAT, and GSH [192,193]. These effects are primarily mediated through the activation of the Nrf2 signaling pathway, which plays a crucial role in cellular defense against oxidative stress [194,195].

Moreover, caffeic acid has been observed to inhibit the activation of nuclear factor kappa B NF-kB, a key transcription factor involved in the inflammatory response [196]. This inhibition reduces the secretion of pro-inflammatory cytokines, such as interleukin-8 (IL-8), thereby mitigating intestinal inflammation [197]. Additionally, caffeic acid modulates the phosphoinositide 3-kinase/protein kinase B (PI3K/Akt) and mitogen-activated protein kinase (MAPK) signaling pathways, including extracellular signal-regulated kinases (ERK), c-Jun N-terminal kinase (JNK), and p38 MAPK [198]. These pathways are integral to the regulation of cellular responses to oxidative stress and inflammation [199].

Collectively, these findings suggest that caffeic acid exerts protective effects against oxidative stress and inflammatory conditions in intestinal epithelial cells, highlighting its potential therapeutic applications in managing intestinal inflammation and related disorders [200].

Through these antioxidant and anti-inflammatory mechanisms, caffeic acid has also been identified as an epigenetic modulator.

Recent research highlights its capacity to influence DNA methylation and histone modifications, two key mechanisms controlling gene expression without altering the underlying DNA sequence [201]. Specifically, caffeic acid has been shown to inhibit DNA methyltransferase (DNMT) activity, potentially reversing abnormal gene silencing associated with chronic diseases such as cancer and inflammatory disorders. Moreover, caffeic acid may affect histone acetylation and methylation, thereby further regulating genes involved in oxidative stress responses, inflammation, and cellular homeostasis.

These findings show that caffeic acid protects intestinal cells not only by reducing oxidative stress and inflammation but also by helping restore healthy gene activity, making it a promising natural compound for managing intestinal disorders.

P-Coumaric acid, a naturally occurring hydroxycinnamic acid found in foods like red wine and balsamic vinegar, exhibits significant antioxidant and anti-inflammatory properties [202,203]. In vitro studies using lipopolysaccharide (LPS)-simulated RAW 264.7 macrophages have demonstrated that treatment with p-coumaric acid at concentration ranging from 10 to 100 μg/mL effectively reduces the production of nitric oxide (NO) and suppresses the expression of pro-inflammatory mediators such as inducible nitric oxide synthase (iNOS) and cyclooxygenase 2 (COX-2) at both mRNA and protein levels [151,204].

The anti-inflammatory effects of p-coumaric acid are primarily mediated through the inhibition of the nuclear factor kappa B (NF-kB) signaling pathway. Specifically, p-coumaric acid prevents the phosphorylation and subsequent degradation of IκBα, an inhibitor of NF-kB, thereby blocking the translocation of the NF-kB p65 subunit into the nucleus and reducing the transcription of pro-inflammatory genes [100].

Additionally, p-coumaric acid modulates the mitogen-activated protein kinase (MAPK) pathways by inhibiting the phosphorylation of extracellular signal-regulated kinase (ERK1/2) and c-Jun N-terminal kinase (JNK), which are crucial in the expression of inflammatory mediators [205].

Beyond its effects on macrophages, p-coumaric acid has been shown to protect against atherosclerosis. In studies involving THP-1 macrophages treated with oxidized low-density lipoprotein (ox-LDL) and LPS, p-coumaric acid at 20 μM significantly inhibited lipid accumulation and foam cell formation. This effect is attributed to the upregulation of cholesterol efflux-related genes, such as ATP binding cassette transporter A1 (ABCA1), liver X receptor alpha (LXRα), and peroxisome proliferator-activated receptor gamma (PPARγ), and the downregulation of lipid uptake related genes, including lecithin-like oxidized low-density lipoprotein receptor-1 (LOX-1), cluster of differentiation 36 (CD36), and scavenger receptor class A1 (SR-A1). Furthermore, p-coumaric acid suppressed the expression of NF-kB, COX-2, tumor necrosis factor-alpha (TNF-α), and interleukin-6 (IL-6), suggesting its potential to mitigate the inflammatory response associated with atherosclerosis. These findings suggest that p-coumaric acid holds promise as a therapeutic agent in managing oxidative stress-related inflammatory diseases, such as atherosclerosis, by modulating key. Inflammatory signaling pathways and promoting cholesterol homeostasis [206].

Anacardic acid, a phenolic lipid predominantly found in cashew nuts (Anacardium occidentale) and mango seeds, has gained attention for its potent neuroprotective properties, particularly in the context of neurodegenerative diseases such as Parkinson’s and Alzheimer’s [207].

In a study by Augusto et al. (2020) [208], purified anacardic acid (AAs) were administered at a dose of 50 mg/kg in a rotenone-induced mouse model of Parkinson’s disease. The treatment led to a significant reduction in lipid peroxidation and NO levels, alongside an increase in the glutathione (GSH)/oxidized glutathione (GSSH) ratio in both the substantia nigra and striatum regions of the brain. These changes indicate a restoration of redox balance and mitigation of oxidative stress-induced neuronal damage [208].

Furthermore, the study observed that AAs attenuated nuclear kappa B (NF-kB) activation and reduced the expression of pro-inflammatory cytokines, such as interleukin-1β (IL-1β). This anti-inflammatory effect was complemented by the downregulation of matrix metalloproteinase-9 (MMP-9) and glial fibrillary acidic protein (GFAP), markers of neuroinflammation and astrogliosis.

In addition to its neuroprotective and anti-inflammatory effects, anacardic acid also shows potential as a natural modulator of gene activity. Recent studies suggest that it can block the activity of histone acetyltransferases (HATs), particularly p300/CBP, enzymes that normally loosen chromatin structure to activate gene expression [209]. By limiting histone acetylation, anacardic acid may help turn down the expression of pro-inflammatory genes and other harmful pathways involved in neurodegenerative diseases. This ability to influence gene regulation adds another important layer to its protective action, complementing its antioxidant and anti-inflammatory effects. Together, these findings highlight anacardic acid as a promising natural compound that works on multiple levels to protect neurons and slow the progression of neurodegeneration. Allyl mercaptan, a sulfur-containing compound derived from garlic (*Allium sativum*), has been shown to enhance the activity of key antioxidant enzymes such as superoxide dismutase (SOD) and catalase (CAT), while also reducing intracellular reactive oxygen species (ROS) levels in HUVEC cells treated at a concentration of 15 μM [210]. This antioxidant enhancement helps preserve redox homeostasis in vascular endothelial cells [211].

### 3.4. Organosulfur Compounds

In addition to its antioxidant effects, allyl mercaptan also inhibits the expression of adhesion molecules, such as ICAM-1 and VCAM-1. It reduces vascular inflammation by preventing NF-kB nuclear translocation, a key event in endothelial activation. Although the precise molecular pathway remains under investigation, some studies have demonstrated that garlic-derived compounds, such as diallyl disulfide and diallyl trisulfide, may act synergistically or more potently by inhibiting NF-kB [212].

These findings support the therapeutic potential of allyl mercaptan as a candidate for endothelial protection and cardiovascular disease prevention, particularly in the context of oxidative stress and inflammation [213].

Moreover, beyond its antioxidant and anti-inflammatory action, allyl mercaptan has been reported to exert epigenetic regulatory effects. According to findings on carcinogenesis [214], allyl mercaptan can inhibit DNMT activity, leading to hypomethylation of promoter regions in key genes involved in cell regulation, such as p21 WAF1. This epigenetic modulation reactivates tumor suppressor pathways and contributes to the regulation of cellular proliferation and apoptosis. Therefore, the protective role of allyl mercaptan in vascular endothelial cells may not only involve redox homeostasis and inflammation suppression but also the regulation of gene expression through epigenetic modifications, highlighting its complex mechanism of action in maintaining cardiovascular health.

### 3.5. Terpenoids

Artemisinin, a sesquiterpene lactone extracted from Artemisia annua, has shown strong antioxidant, anti-inflammatory, and anticancer properties [215,216]. In HeLa cells treated with 50 μM artemisinin, studies have demonstrated a significant reduction in ROS and enhanced expression of antioxidant enzymes, such as heme oxygenase-1 (HO-1), via activation of the Nrf2 signaling pathway [217,218].

In addition to its antioxidant effects, artemisinin effectively inhibits NF-kB activation, leading to downregulation of pro-inflammatory cytokines such as IL-1β, TNF-α, and COX-2 [219,220]. These molecular effects contribute to the compound’s ability to suppress the tumor microenvironment’s inflammation and modulate immune responses.

Artemisinin also exerts pro-apoptotic activity, inducing loss of mitochondrial membrane potential, caspase activation, and cell cycle arrest in various cancer cell lines, including breast, cervical, and colorectal cancers [221]. Moreover, its antiangiogenic properties, achieved through inhibition of VEGF-mediated endothelial cell migration and proliferation, make it a valuable candidate in anti-tumor therapy, particularly for limiting tumor neovascularization [222].

Due to its multimodal activity profile—antioxidant, anti-inflammatory, apoptotic, and anti-angiogenic—artemisinin is increasingly regarded as a promising adjunct in oncological treatment and is currently under investigation in both preclinical and early-phase clinical studies [223].

Geraniol, a monoterpene found in rose and citronella essential oils, reduces ROS levels and increases GSH activity in RAW 264.7 macrophages (50 μM). Inhibiting NF-kB decreases NO, IL-6, and TNF-α [224]. Geraniol also modulates the PI3K/Akt and MAPK pathways, with potential applications in managing chronic inflammatory and metabolic disorders [225].

Recent research has uncovered an emerging role for geraniol in epigenetic regulation. A 2025 study demonstrates that geraniol can influence gene expression by modulating DNA methylation and histone acetylation, processes essential for controlling gene expression [226]. Specifically, geraniol was found to inhibit the expression of DNA methyltransferases (DNMTs) and histone deacetylases (HDACs), enzymes that play key roles in maintaining aberrant gene silencing in inflammation and metabolic dysfunction. Through this action, geraniol may help restore normal gene expression patterns, further supporting its protective effects at the molecular level. This evidence suggests that geraniol supports health by reducing oxidative stress and inflammation while also helping to restore healthy gene activity, making it a promising natural option for preventing chronic diseases.

Various classes of bioactive compounds, including flavonoids (hesperidin, phloretin, silybin), carotenoids (lycopene), phenolic acids (caffeic acid, p-coumaric acid, anacardic acid), sulfur-containing molecules (allyl mercaptan), terpenes (geraniol), and sesquiterpene lactones (artemisinin), have demonstrated strong antioxidant and anti-inflammatory activities in multiple in vitro studies.

Most of these phytochemicals enhance the activity of endogenous antioxidant enzymes such as SOD, CAT, and GSH, while reducing lipid peroxidation and ROS accumulation. These mechanisms contribute to cellular protection against oxidative damage.

Additionally, several compounds, such as hesperidin, lycopene, artemisinin, and silybin, exhibit antiproliferative effects by inhibiting NF-kB signaling and suppressing pro-inflammatory mediators, including TNF-α, IL-6, COX-2, and iNOS. These bioactive compounds support potential roles as chemopreventive and therapeutic agents in inflammation-associated disease, including cancer, cardiovascular, and neurodegenerative disorders.

Moreover, several compounds, such as hesperidin, lycopene, artemisinin, and silibinin, exhibit antiproliferative effects by inhibiting NF-κB signaling and suppressing pro-inflammatory mediators, including TNF-α, IL-6, COX-2, and iNOS. These bioactivities support their potential roles as chemopreventive and therapeutic agents in inflammation-associated diseases, including cancer, cardiovascular, and neurodegenerative disorders.

Interestingly, some phytochemicals can epigenetically regulate NRF2 levels, which act as a master regulator of the antioxidant response. As such, the coupling of NRF2 to antioxidant response element sequences in gene promoters results in mechanisms of antioxidant detoxification. Yates et al. Phytochemicals documented to modulate NRF2 signaling act by reversing hypermethylated states in the CpG islands of NFE2L2 or Nfe2l2, via the inhibition of DNA methyltransferases (DNMTs) and histone deacetylases (HDACs), through the induction of ten-eleven translocation (TET) enzymes, or by inducing miRNA to target the 3′-UTR of the corresponding mRNA transcripts. To date, fewer than twenty phytochemicals have been reported as NRF2 epigenetic modifiers, including curcumin, sulforaphane, resveratrol, reserpine, and ursolic acid. Bhattachaarjee et al. [227] provided a broader view of the action of phytochemicals on a broad array of disorders, including cancer and chronic diseases. A more focused insight is provided [228], which focuses on natural compounds acting as epigenetic regulators in diabetes mellitus, with curcumin, resveratrol, sulforaphane, genistein, and quercetin identified as major actors.

Naturally occurring phytochemicals, such as those mentioned above, along with other well-studied compounds like resveratrol [229], quercetin [230], and apigenin [231], are commonly used in herbal extracts and have potential as complementary therapies for managing inflammatory diseases, cancer, and other chronic conditions.

When these bioactive compounds are consumed from natural sources, they are typically present alongside other related phytochemicals. This combination allows them to act on multiple regulatory pathways simultaneously [232]. Additionally, these natural compounds can be combined with synthetic medicines to create more complex therapeutic strategies that target several disease mechanisms simultaneously [233,234].

### 3.6. Synergistic Effects of Phytochemical Combinations

Among the various phytochemicals examined, the combination of genistein and lycopene emerges as one of the most promising candidates for exploring synergistic interactions between natural compounds. Both exhibit potent antioxidant, anti-inflammatory, and epigenetic modulatory properties, yet they act through complementary molecular pathways that may synergize to enhance their bioactivity and therapeutic potential.

Genistein, a soy-derived isoflavone, functions as a multifaceted modulator of cellular signaling. It reduces oxidative stress by stimulating antioxidant enzymes (SOD, CAT, GPx) via the Nrf2 pathway, while simultaneously suppressing pro-inflammatory mediators by inhibiting NF-κB signaling [235]. At the epigenetic level, genistein inhibits DNA methyltransferases (DNMTs) and histone deacetylases (HDACs), leading to reactivation of silenced tumor suppressor genes such as *p16*, *RASSF1A*, and *BRCA1* [236]. This dual activity—antioxidant and epigenetic—positions genistein as a model nutraceutical for cancer prevention and modulation of chronic disease [237].

Lycopene, a carotenoid abundant in tomatoes and other red fruits, is characterized by its exceptional lipophilic antioxidant capacity [238]. It effectively quenches singlet oxygen and scavenges reactive oxygen species (ROS), protecting membrane lipids from peroxidation [239]. Lycopene also modulates transcription factors involved in inflammation and proliferation, including AP-1, STAT3, and COX-2, thus exerting anti-inflammatory and antiproliferative effects [240]. Moreover, lycopene has been reported to indirectly influence epigenetic processes, such as modulating DNMT activity and microRNA expression [153].

The synergistic rationale for combining genistein and lycopene lies in their complementary mechanisms: genistein acts primarily at the nuclear level, regulating chromatin and gene expression, while lycopene stabilizes redox and membrane integrity, reducing the oxidative triggers that often initiate epigenetic aberrations [241,242].

This cross-interaction between epigenetic reprogramming and oxidative balance could yield enhanced protective effects against carcinogenesis, metabolic inflammation, and age-related disorders [243]. In addition, lycopene’s lipid-soluble nature may improve genistein’s bioavailability by facilitating its absorption and protecting it from intestinal degradation, addressing one of the main challenges in translating genistein’s benefits to clinical settings [244,245].

Emerging evidence from preclinical studies supports the combined use of polyphenols and carotenoids as a novel strategy in nutraceutical formulations [246,247,248]. However, studies specifically investigating the synergy between genistein and lycopene remain limited. Future research should employ co-administration models in vitro and in vivo, focusing on oxidative stress–related diseases such as breast, liver, or prostate cancer. Key endpoints should include measurements of oxidative stress markers, inflammatory cytokine profiles, DNMT/HDAC activity, and downstream gene expression changes associated with cellular homeostasis [249,250].

Furthermore, advanced delivery systems, such as nanoemulsions, liposomes, or polymeric nanoparticles, could be explored to optimize their pharmacokinetic profiles and ensure co-delivery to target tissues. The integration of gut microbiota analysis in these models may also help elucidate how microbial metabolism influences the combined bioactivity of genistein and lycopene [251,252,253].

Altogether, this phytochemical pair exemplifies how strategic combinations of compounds can act on distinct yet interconnected molecular networks—redox regulation, inflammation, and epigenetic remodeling—paving the way for more effective dietary interventions and adjuvant therapies in chronic diseases and cancer prevention.

### 3.7. Challenges and Strategies for Clinical Translation

Despite the promising antioxidant, anti-inflammatory, and epigenetic-modulating capacities of specific phytochemicals, translating these findings from preclinical to clinical applications remains a significant challenge.

The flavonoids (e.g., hesperidin, genistein, phloretin, and silybin) exhibit strong biological effects in vitro but have low oral bioavailability due to poor solubility and extensive first-pass metabolism [254,255]. For instance, hesperidin and genistein require transformation by gut microbiota into more active metabolites, introducing high interindividual variability. Strategies such as glycosylation modification, nanoencapsulation, and phospholipid complexes have been investigated to improve their absorption and stability [256].

Lycopene (carotenoid) exhibits potent antioxidant and anti-inflammatory activity; however, it is lipophilic and unstable, limiting its systemic availability [257]. Current approaches include liposomal formulation and emulsified delivery systems to enhance its stability and intestinal uptake [258].

Phenolic compounds are rapidly metabolized and eliminated, resulting in a short plasma half-life and reduced clinical efficacy. Moreover, their metabolism is significantly modulated by gut microbiota composition, which can differentiate between individuals. To overcome this, prodrug strategies and targeted delivery systems have been proposed, though evidence remains scarce [259].

Allyl mercaptan (organosulfur compounds) derived from garlic is strongly affected by gut metabolism and instability in circulation. Clinical translation requires stabilized formulation or co-administration with other garlic-derived sulfur compounds to preserve its bioactivity [260].

Artemisinin (a terpenoid) has potent anti-inflammatory and anticancer activity, but rapid clearance and poor water solubility limit its clinical utility. Semi-synthetic derivatives and nanocarrier systems are being explored to overcome these pharmacokinetic barriers [261,262]. Geraniol also suffers from limited bioavailability, and lipid-based carrier encapsulation has been proposed as a solution [254,263].

While small-scale clinical trials with flavonoids and carotenoids have shown beneficial effects on oxidative stress and metabolic regulation, results remain heterogeneous and not sufficient for clinical recommendations [264]. For other compounds reviewed (phloretin, caffeic acid, p-coumaric acid, anacardic acid, allyl mercaptan, geraniol), clinical evidence is very limited or absent, emphasizing the urgent need for rigorous, well-designed clinical trials. This gap is consistent with previous findings on the anticancer and epigenetic modulation activity of natural compounds [265].

## 4. Conclusions and Future Perspectives

The preventive potential of phytochemicals is a rapidly growing area of research, with in vitro and in vivo studies demonstrating their ability to simultaneously target multiple cellular and molecular pathways, thereby enhancing biological effects. These natural bioactive compounds are increasingly recognized for their roles in promoting health and preventing chronic disease. The majority of chronic diseases are characterized by early epigenetic dysregulation, which may disrupt normal patterns of gene expression. Intrinsic factors such as aging and genetic background, along with extrinsic environmental factors (e.g., diet, pollution, radiation, stress), can significantly influence epigenetic mechanisms, ultimately impacting human health.

The reversibility of epigenetic modifications and the ability to reset cellular homeostasis through compounds with epigenetic modulator capacity have opened new avenues for prevention interventions, particularly in aging, cancer, cardiovascular, and metabolic disorders. Targeting these reversible changes with dietary bioactive compounds offers a promising approach to prevent diseases where early epigenetic abnormalities play a key role.

This review emphasizes that certain phytochemicals, including flavonoids, which possess both antioxidant and anti-inflammatory properties, can directly or indirectly inhibit the aberrant activities of epigenetic regulators. The underlying molecular mechanisms of phytochemicals’ multifaceted actions are incompletely understood. Still, their capacity to act through various epigenetic pathways, including DNA methylation, histone modifications, and chromatin remodeling, is likely to impact a range of cellular processes.

The translation of preclinical data into preventive medicine is a challenging process that requires rigorous clinical trials while accounting for individual variability in the gut microbiota and its impact on phytochemical metabolism. Long-term trials, which should consider the synergistic effects of dietary phytochemicals, are also needed to evaluate overall efficacy and safety in humans, as the diet provides a combination of phytochemicals with specific cellular and organ-level actions.

Despite promising laboratory results, a key challenge remains the low bioavailability of many phytochemicals, which limits their therapeutic translation. Strategies such as nanocarrier delivery systems, liposomal encapsulation, structural modification of parent compounds, and co-administration with bioenhancers are currently being investigated to improve absorption stability and tissue targeting.

Another critical aspect is the inter-individual variability in gut microbiota composition, which profoundly affects phytochemical metabolism and biological activity. Personalized approaches, including microbiome profiling, may be required in the further design of trials to better capture this variability.

While preclinical evidence is extensive, clinical studies remain limited and often heterogeneous. Some small-scale trials have shown beneficial effects of polyphenols (e.g., genistein) on biomarkers of oxidative stress, inflammation, or metabolic regulation, but larger, standardized, placebo-controlled trials are lacking.

Therefore, future clinical trials should be designed with adequate sample sizes, stratification of individuals by microbiota/metabolic phenotype, and long-term follow-up, while considering dietary context and potential synergistic effects with conventional therapies.

## Data Availability

No new data were created or analyzed in this study. Data sharing is not applicable to this study.
